# Internal capsule: The homunculus distribution in the posterior limb

**DOI:** 10.1002/brb3.629

**Published:** 2017-02-06

**Authors:** Cheng Qian, Fei Tan

**Affiliations:** ^1^ Department of NeurologyShengjing Hospital of China Medical UniversityShenyangChina

**Keywords:** Homunculus, Infarction, internal capsule, posterior limb of the internal capsule

## Abstract

**Introduction:**

In our experience, sometimes, the symptom of patients who suffered from infarction in internal capsule (IC) do not necessarily fit the classical fiber distribution. This study aims to explain this phenomenon.

**Methods and Materials:**

A total of 34 patients with infarction lesions in the IC were included in this study, according to the clinical symptom, divided into three groups, group A (more severe weakness of the foot than the hand), group B (more severe weakness of the hand than the foot) and group C (equal weakness of hand and foot), and group Y (with facial nerve paresis) and group N (without facial nerve paresis). Measurements included the length ratio and the angle degree of infarction lesions compared with the posterior limb of the IC (PLIC).

**Results:**

The length ratio of infarction lesions is significant difference between group A and group B (*p* = .027), the angle degree of infarction lesions is significant difference between group Y and group N (*p* = .038).

**Conclusion:**

From our results, we can conclude that the hand fibers are located laterally to foot fibers in the short axis of the posterior limb of the IC, and the face fibers are located in the premedial part of the posterior limb of the internal capsule.

## 1 Introduction

The internal capsule (IC) is an important structure in the brain that consists of descending and ascending fibers tracts. It is generally thought that the two main descending fibers tracts, the corticobulbar tract and the corticospinal tract, descend separately through the genu (Charcot, 1883) and through the anterior third of the posterior limb of the internal capsule (PLIC) (Dejerine, 1901; Foerster, 1936). In the PLIC, corticospinal tracts are organized along a long axis, meaning that hand fibers are located anteromedial to foot fibers (Bertrand, Blundell, & Musella, 1965; Carpenter, 1983). However, in our experience, some patients suffer from infarction in the IC, and their symptoms do not necessarily fit the classical fiber distribution. This study aims to address this phenomenon by analyzing the infarction lesion and clinical symptoms of patients who suffered acute infarction in the IC.

## 2 Materials and Methods

This article is based on 34 patients who visited the neurological departments of two hospital centers belonging to the Shengjing Hospital of China Medical University between 26/06/2010 and 05/12/2015. Inclusion criteria were as follows: acute infarction, single lesion in the IC, and performed magnetic resonance diffusion‐weighted imaging (MR‐DWI). Exclusion criteria were as follows: presence of any other brain disease, previous paresis, a history of drug dependency, and presence of a psychiatric disorder. The study was approved by the ethics committee of Shengjing Hospital of China Medical University.

### 2.1 MR acquisition

MR images were acquired using MR Philips 1.5T, MR Philips 3.0T or MR GE 3.0T. Diffusion‐weighted imaging (DWI) is a form of MR imaging based upon measuring the random Brownian motion of water molecules within a voxel of tissue and is essential for the diagnosis of acute infarctions.

### 2.2 Data measurements

**Figure 1:**
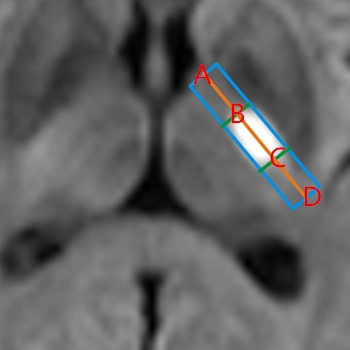
Ratios of “AB/AD” and “AC/AD” “AD” represents the length

The length ratio of infarction lesion compared PLIC: As the Figure 1 shows, four point are marked, “AD” represent the length of PLIC, “BC” represent the length of the infarction lesion, and the length of “AD”, “AB” and “AC” are measured, further, the ratios of infarction lesion compared PLIC, “AB/AD” and “AC/AD” are calculated.

**Figure 2:**
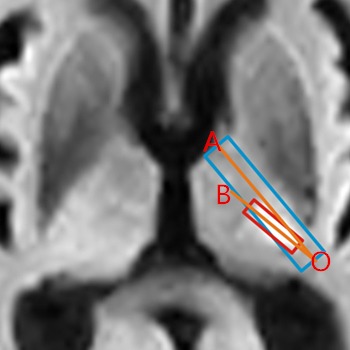
Angle degree of “∠AOB” “AO” represents the perpen

The angle degree of infarction lesion compared PLIC: The Figure 2 shows the measurement of the angle degree of infarction lesion compared PLIC, “∠AOB”, in which “AO” represents perpendicular bisector of the short axis of posterior limb, “BO” represents the long axis of infarction lesion, and if the “BO” is at the thalamus side, the degree is negative, reversely, if the “BO” is at the putamen side, the degree is positive.

All figures were analyzed by Digimizer software.

### 2.3 Statistical analysis

**Figure 3:**
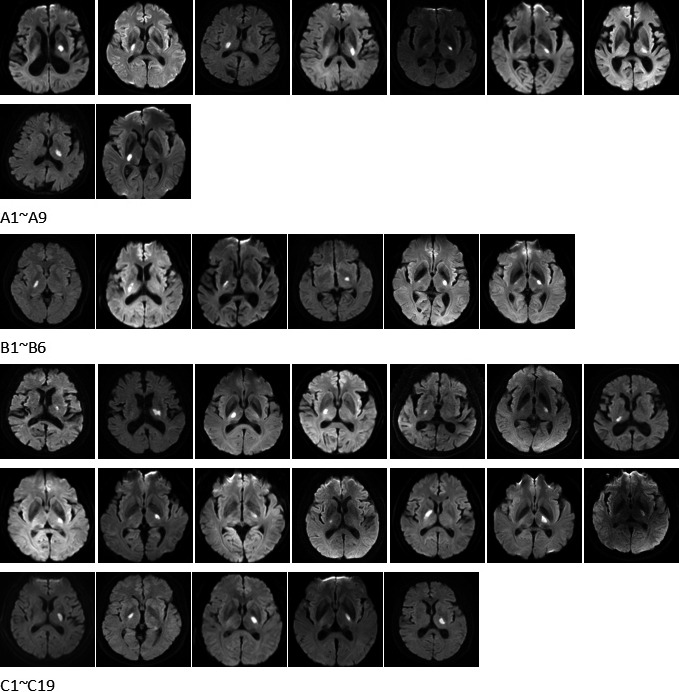
MR images of a total of 34 patients

As Figure 3 shows, 34 patients were divided into three groups, A, B, and C. Group A consisted of patients with a more severe weakness of the foot than the hand, group B represents a more severe weakness of the hand than the foot, and group C represents equal weakness of hand and foot. Furthermore, based on whether the patients suffered from facial nerve paresis, 34 patients were divided into group Y (with facial nerve paresis) and group N (without facial nerve paresis).

Welch's t test was used to compare the mean values of group A, B, C and group Y, N, with *p* < .05 indicating statistical significance, and box‐plots would be presented when there was any statistical significance.

## 3 Results

Table 1 shows demographic and clinical data of the 34 patients.

**Table 1:** Demographic and clinical data of the 34 patients

No.	Age	Sex	Hand^a^	Foot^a^	Face paresis^b^	Tongue paresis^b^	Dysarthria^b^	AB/AD	AC/AD	∠AOB
A1	73	M	5−	4+	0	0	0	0.219	0.619	3.206
A2	59	M	5−	3	0	0	0	0.394	0.812	7.458
A3	56	M	5−	4	0	0	0	0.429	0.873	−6.468
A4	69	M	5	4	0	0	1	0.532	0.901	−5.767
A5	66	F	3−	2−	0	0	0	0.539	0.818	−0.616
A6	91	F	5	4−	0	0	0	0.549	0.945	−4.228
A7	83	M	5	4	0	0	0	0.571	0.8	−20.518
A8	71	F	5−	4−	1	0	0	0.239	0.777	−4.619
A9	41	M	5−	4−	1	1	0	0.553	0.902	−8.037
B1	47	F	1	3−	1	1	0	0.255	0.668	0.459
B2	71	F	3	4	0	0	0	0.263, 0.435^c^	0.81, 0.535^c^	5.752
B3	71	M	3	4	0	1	1	0.388	0.814	5.503
B4	69	F	4−	5−	1	0	1	0.443	0.777	6.866
B5	61	M	3	4	1	0	0	0.6	0.901	25.442
B6	59	M	4	5−	1	0	1	0.483	0.916	−2.392
C1	80	F	5−	5−	0	0	0	0.171	0.508	0.013
C2	84	M	5−	5−	0	0	0	0.189	0.778	−2.445
C3	61	M	5	5	0	0	0	0.428	0.755	−2.961
C4	65	M	5	5	0	0	0	0.434	0.815	5.791
C5	87	F	5	5	0	0	1	0.445	0.669	2.695
C6	69	M	3	3	0	0	0	0.537	0.825	1.35
C7	64	F	5	5−	0	0	0	0.541	0.939	0.075
C8	57	F	5	5	0	0	0	0.548	0.874	0.071
C10	54	M	5	5	0	0	1	0.587	0.906	4.821
C9	56	M	4	4	0	0	0	0.568	0.97	−0.202
C11	56	F	5−	5−	0	0	0	0.672	0.844	−11.694
C12	58	M	5−	5−	1	0	1	0	0.564	7.742
C13	44	M	5−	5−	1	0	1	0.366	0.788	−11.702
C14	41	M	4−	4−	1	1	1	0.407	0.862	0.699
C15	49	M	4	4	1	1	1	0.22	0.81	4.096
C16	53	M	5	5	1	0	1	0.264	0.664	7.53
C17	54	M	5	5	1	0	1	0.308	0.783	3.408
C18	60	M	5−	5−	1	1	1	0.336	0.846	0.352
C19	73	F	4	4	1	1	0	0.406	0.943	−14.306

a The myodynamia of the hand and foot.

b “1” means that patient had symptom, “0” means no.

c B2 had two lesions in the IC, the total length proportion of two lesion was 0.263–0.81, and between 0.435 and 0.535, there was a gap.

**Figure 4:**
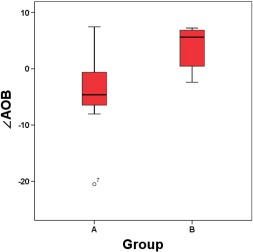
Boxplots of group A and group B

The Figure 4 shows the differences between the angle degree of infarction lesion, “∠AOB”, of group A and group B, and the means of “∠AOB” of group A and group B are −4.399, 3.905, the *p* value is lower than .05, having significant difference. While the means of “AB/AD” and “AC/AD” have no significant difference according to the *p* value (Table 2).

**Table 2:** Group A and group B

	A *N*, Mean SD	B^a^ *N*, Mean SD	*p* Value
AB/AD	9, 0.447 0.137	6, 0.405 0.133	.543
AC/AD	9, 0.827 0.096	6, 0.814 0.090	.849
∠AOB	9, −4.399 7.842	6, 7.842 9.746	.027

a B2 is consider as one lesion, the AB/AD is 0.263, the AC/AD is 0.81.

**Figure 5:**
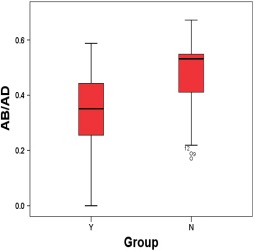
Boxplots of group Y and group N

Meanwhile the Figure 5 shows the differences between the length ratio of infarction lesion of group Y and group N, and the means of “AB/AD” of group Y and group N are 0.348, 0.46, the *p* value is lower than .05, having significant difference, while the means of “AC/AD” and”∠AOB” have no significant difference according to the *p* value (Table 3).

**Table 3:** Group N and group Y

	N *N*, Mean SD	Y *N*, Mean SD	*p* Value
AB/AD	19^a^, 0.46 0.14	14, 0.349 0.154	.038
AC/AD	19, 0.814 0.115	14, 0.8 0.11	.729
∠AOB	19, −1.259 6.658	14, 1.11 9.83	.415

a B2 is excluded.

## 4 Discussion

The difference between “∠AOB” of group A and group B suggests that the hand fibers locate laterally to foot fibers in the short axis of PLIC. And the mean “AB/AD” of group N, 0.46, suggests that the face fibers locate in the anteromedial portion of the PLIC. In the other way, the 0.348, the mean “AB/AD” of group Y suggests that face fibers cannot just locate in the anterior third of PLIC, let alone the genu of IC.

In brief, there are two main findings: first, hand fibers seem to be located laterally to foot fibers in the short axis of PLIC; second, face fibers are likely located in the anteromedial portion of the posterior limb of the IC.

Neither of these two findings fits the classical distribution of descending fibers in the PLIC, supporting previous studies casting doubt on the classical anatomy (Englander, Netsky, & Adelman, 1975; Kretschmann, 1988; Yagishita, Nakano, Oda, & Hirano, 1994). Holodny, Gor, Watts, Gutin, and Ulu (2005) demonstrated that the corticospinal tracts are located in the posterior third quarter of the PLIC and that hand fibers are located anterolateral to foot fibers. Yim et al. (2013) suggested that the corticobulbar tracts pass through the median of the PLIC instead of the genu. Moreover, they calculated the ratio of the lesion compared with the PLIC and found that the most overlapping area was the median of the PLIC.

However, a recent study (Duerden, Finnis, Peters, & Sadikot, 2011) investigating the somatotopic organization and probabilistic mapping of motor responses in the IC found a contrasting result. Using electrophysiology, the authors found that face fibers were located anteromedial to hand responses, while foot fibers lied posterolateral to the hand representation. Interestingly, 45% of total leg responses co‐occurred with arm responses. On the one hand, this indicates overlap between leg fibers and hand fibers in the IC; on the other hand, this might be the reason why their result conflicts with both Holodny et al. (2005) study and this study. Or perhaps, they are all right, the difference just means the different level of the structure of IC. It is well‐known that somatotopy of hand is located lateral to the foot in the centrum semiovale (Seo, Chang, & Jang, 2012; Zolal et al., 2012), but in the brainstem, the distribution is reversed, meaning that somatotopy of hand is located medial to the foot (Hong, Son, & Jang, 2010; Kwon, Hong, & Jang, 2011). So the rotation does have to take place in the median between precentral gyrus and brain stem, and IC is just the median. However, most of our patients had lesions in the cranial dorsal part of the internal capsule, where the pyramidal tract may not be completely rotated yet.

## 5 Limitations

This study has some limitations. First, the sample size was relatively small. Second, the measurement of the angle degree of “∠AOB” has several human errors, as the long axis of the lesion was difficult to draw, especially the line of OB. Third, patient images were obtained using different MRI scanners, 1.5 T and 3.0 T, leading to potential differences in infarction margins between the two MRI machines. Fourth, IC images of patients were not obtained in the same horizontal section, potentially changing the relative location of the genu of the IC (Axer & Keyserlingk, 2000).

## 6 Conclusion

This study suggests two main findings: first, hand fibers seem to be located laterally to foot fibers in the short axis of the PLIC; second, face fibers are likely located in the anteromedial portion of the posterior limb of the IC. Given the importance of the structure, even small lesions of the IC can lead to severe outcomes, emphasizing the need for further studies to provide novel insights in fiber distributions.
